# American Medical Association

**Published:** 1875-07-01

**Authors:** 


### AMERICAN MEDICAL ASSOCIATION.

THE address of Dr. Austin Flint, of New York, as chairman of the Section on Practical Medicine, Materia Medica, and Physiology, was read in the general session of the Association on the second day of the meeting, and was listened to with great attention. Dr. Flint referred very briefly to such topics in Materia Medica and Physiology as have been the subject of special investigation during the past year, including the localization of motor centres in the periphery of the brain, the consumption of alcohol in the living animal system, and the transfusion of blood.

Concerning the latter topic he stated that, though many experiments had been performed, both in the transfusion of the blood of animals of the same and of different species, yet the results did not justify the establishment of any practical conclusions concerning the remedial value of the practice in the treatment of disease. Although he alluded somewhat at length to the recent experiments of the late Dr. Anstie, and others, concerning the effects of alcohol and its retention in the system to the extent of six hundred grains per day, he gave no explanation of the alleged fact. It is well known that Dr. Anstie in his latest experiments on animals, claimed to prove that only a small part of the alcohol introduced into the system was eliminated again in the form of alcohol; but that an amount equal to six hundred grains of pure alcohol was capable of being taken daily and consumed in the system of an adult man, without re-appearing in the excretions or inducing any toxemic or deleterious effects. And he stated that if alcohol to the extent of six hundred grains could be thus consumed in the system without either affording material for nutrition or the generation of force, it would constitute one of the most singular facts in the domain of science. Such a statement is evidently founded on the assumption that all substances taken into the system and retained, must either be added to the tissues as food, or enter into such combinations as to generate force. But is such an assumption justified by the known facts concerning the action of medicinal agents in the living system? Is it not true that the preparations of lead, mercury, arsenic, and many other poisons, may be taken in small doses, repeated daily, and much of them be retained in the system, without inducing direct toxemic effects? We had supposed it to be a well known fact in chemistry, that some substances were capable of exerting a catalytic action, in some instances by their presence retarding the combination of other substances, and in others increasing it. And we see no reason why alcohol, by its affinity for the albumen and water of the blood and tissues, may not be held in the human body in small quantities without being eliminated with sufficient rapidity to be readily detected in the excretions, and instead of being itself appropriated as nutritive material, simply act as a retarder, both of atomic changes and of the organic forces. Indeed, the playing of such a part in the animal economy, by alcohol, is in strict accordance with all the results of experience. After noticing briefly the chief topics concerning which recent investigations had been made, Dr. Flint devoted an important part of his address to the subject of the natural history of crime, with special reference to its connection with the physiological and pathological condition of the criminal. He urged the adoption of measures for a systematic investigation of the subject; and at the close of his address he was requested, by a vote of the Association, to continue his researches in this direction and embody the results in a future report.

